# A Conserved Cysteine Motif Is Critical for Rice Ceramide Kinase Activity and Function

**DOI:** 10.1371/journal.pone.0018079

**Published:** 2011-03-31

**Authors:** Fang-Cheng Bi, Quan-Fang Zhang, Zhe Liu, Ce Fang, Jian Li, Jian-Bin Su, Jean T. Greenberg, Hong-Bin Wang, Nan Yao

**Affiliations:** 1 State Key Laboratory of Biocontrol, School of Life Sciences, Sun Yat-sen University, Guangzhou, China; 2 Department of Molecular Genetics and Cell Biology, The University of Chicago, Chicago, Illinois, United States of America; Iwate University, Japan

## Abstract

**Background:**

Ceramide kinase (CERK) is a key regulator of cell survival in dicotyledonous plants and animals. Much less is known about the roles of CERK and ceramides in mediating cellular processes in monocot plants. Here, we report the characterization of a ceramide kinase, *OsCERK*, from rice (*Oryza sativa* spp. *Japonica* cv. Nipponbare) and investigate the effects of ceramides on rice cell viability.

**Principal Findings:**

*OsCERK* can complement the Arabidopsis CERK mutant *acd5*. Recombinant OsCERK has ceramide kinase activity with Michaelis-Menten kinetics and optimal activity at 7.0 pH and 40°C. Mg^2+^ activates OsCERK in a concentration-dependent manner. Importantly, a CXXXCXXC motif, conserved in all ceramide kinases and important for the activity of the human enzyme, is critical for OsCERK enzyme activity and *in planta* function. In a rice protoplast system, inhibition of CERK leads to cell death and the ratio of added ceramide and ceramide-1-phosphate, CERK's substrate and product, respectively, influences cell survival. Ceramide-induced rice cell death has apoptotic features and is an active process that requires both *de novo* protein synthesis and phosphorylation, respectively. Finally, mitochondria membrane potential loss previously associated with ceramide-induced cell death in *Arabidopsis* was also found in rice, but it occurred with different timing.

**Conclusions:**

*OsCERK* is a *bona fide* ceramide kinase with a functionally and evolutionarily conserved Cys-rich motif that plays an important role in modulating cell fate in plants. The vital function of the conserved motif in both human and rice CERKs suggests that the biochemical mechanism of CERKs is similar in animals and plants. Furthermore, ceramides induce cell death with similar features in monocot and dicot plants.

## Introduction

Sphingolipids are key structural components of membranes and important signal molecules for cell growth, cell death, embryogenesis and development [1], [2], [3]. Sphingolipid metabolism and functions have been intensively researched in animals and yeast. Sphingolipids and their metabolic intermediates, including ceramide, sphingosine, sphingosine-1-phosphate, and ceramide-1-phosphate (C1P), are significant bioactive molecules in a variety of biological processes [4]. Ceramide, which is the central core lipid in the metabolism of sphingolipids, mediates cell cycle events, differentiation, senescence, necrosis, proliferation and apoptosis [5]. Sphingosine, a metabolite of ceramide, exerts pleiotropic effects on protein kinases and other targets in animals, including regulating the actin cytoskeleton, endocytosis, cell cycle and apoptosis [3], whereas sphingosine-1-phosphate is intimately involved in cell motility, cell survival, cell proliferation and inflammation [1], [3]. C1P also has important roles in mediating cell survival, cell proliferation and inflammation [1], [3]. Moreover, the dynamic balance between levels of ceramides and C1P is critical for cell survival in the model dicot plant *Arabidopsis thaliana* and in human cells [1], [6]. In *Arabidopsis*, loss of ceramide kinase (CERK) activity due to mutation of the *ACCELERATED CELL DEATH 5* (*ACD5*) gene results in spontaneous cell death late in development that correlates with the accumulation of ceramides [6].

In animals, CERK activity, first identified in brain synaptic vesicles, is calcium dependent [7]. Human CERK has high specificity for substrate recognition of ceramide [8], [9]. The pleckstrin homology (PH) domain of CERK is indispensable for its activity and acts as a regulator of its targeting [10] and subcellular localization [11]. A cluster of cysteines, C(347)XXXC(351)XXC(354), is important for CERK enzyme activity [12].

In plants, sphingolipids are also major components of plant membranes. Several enzymatic steps related to sphingolipid metabolism in plants are similar to those found in mammals [13]. These include serine palmitoyltransferase [14], [15], [16], ceramide kinase [6], neutral ceramidase [17], LCB kinase [18], [19], [20], LCB phosphatase [20], [21], LCB C-4 hydroxylase [22], fatty acyl α-hydroxylase [23], sphingolipid-Δ8(*E/Z*)-desaturase [24], sphingolipid-Δ4(*E*)-desaturase [25], sphingosine-1-phosphate lyase [26], [27] and inositolphosphorylceramide synthase [28]. However, the function and regulation of these activities in plants still remain largely unknown.

Ceramide is a critical sphingolipid metabolite involved in the regulation of programmed cell death (PCD) in plants [6], [28], [29]. Exogenous ceramides, the substrate of CERK, regulate the effects of the fungal toxin AAL in tomato leaves [30]. Arabidopsis *acd5* mutant plants show excessive cell death and permit enhanced pathogen growth when infected by *Pseudomonas syringae*, indicating that ACD5 plays an important role in controlling disease susceptibility [31]. Moreover, short chain ceramides reduce mitochondrial membrane potential and cause subsequent cytochrome c release in *Arabidopsis* [32].

In monocots, little is known about the roles of ceramides and the enzymes of the sphingolipid pathway. In this paper, we report the cloning of a ceramide kinase in rice and demonstrate that *OsCERK* is a *bona fide* ceramide kinase that can completely rescue *acd5* mutant phenotypes, including spontaneous cell death, susceptibility to bacterial infection, reduced CERK activity and elevated *PR1* expression. We show that a conserved CXXXCXXC motif found in all CERKs is vital to OsCERK function. Finally, we show that ceramide-induced cell death features and antagonism by C1P are similar in rice and *Arabidopsis*, indicating a conserved role for ceramide and ceramide kinase in monocots and dicots.

## Results

### Cloning and phylogenetic analysis of OsCERK

**Figure 1:**
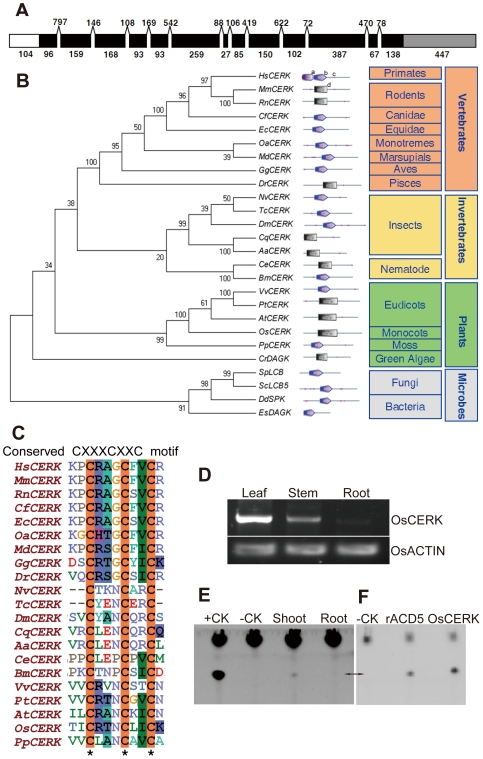
Sequence analysis and expression of rice ceramide kinase (*OsCERK*) *in planta*. (A) Schematic representation of the sequence organization of *OsCERK*. The sizes of the 5′-UTR (white), exons (black) and 3′-UTR (grey) are indicated by numbers below the boxes. The sizes of introns are shown above. (B) Phylogenetic analysis of CERKs and the related enzymes sphingosine kinase (SPK)s, and diacylglycerol kinase (DAGK)s. The architecture of each gene was obtained from SMART using the architecture analysis function, and conserved domains (letters a–d) are indicated as follows: a, Pleckstrin homology domain (PH domain; accession number SM00233); b, Diacylglycerol kinase catalytic domain (DAGKc; SMART accession number SM00046); c, segments of low compositional complexity determined by the program; d, diacylgycerol kinase catalytic domain (DAGK_cat; Pfam accession number PF00781). Detailed sequences information is described in Materials and methods. AtCERK is ACD5. (C) CLUSTAL W alignment of CERK from various organisms showing the highly conserved CXXXCXXC motif. (D) RT-PCR expression analysis of *OsCERK* in leaves, stem and roots of 2-week-old seedlings. β-actin was used as a control. (E) CERK activity of rice extracts. Crude plant extracts made from leaves and roots of rice, respectively, were incubated at 40°C for 30 min as described in Materials and methods. The reaction mixture (1 µl) was resolved on a TLC plate. Purified OsCERK recombinant protein was used as the positive control (+CK). For the negative control (−CK) no extract was added. (F) Comparison of recombinant OsCERK and rACD5. The recombinant protein (1.0 µg) was added in 100 µl reaction system, and incubated at 40°C for 30 min. –CK, no recombinant protein was added. Arrows in (E and F) indicated the formed product C6-NBD-ceramide-1-phosphate (C6-NBD-Cer-1P).

Rice harbors a single gene on chromosome 2 (at locus Os02g0656200) predicted to encode a ceramide kinase. The predicted amino acid sequence of this putative rice ceramide kinase (OsCERK) has 48% sequence identity and 64% sequence similarity to the *Arabidopsis* ceramide kinase called ACCELERATED CELL DEATH 5 (ACD5 [6]) and 27.2% sequence identity and 40.8% sequence similarity to CERK from humans (hCERK, Genbank accession number NP_073603 [8]. The full length *OsCERK* is made up of 13 exons, 104 bp 5′-UTR and 447 bp 3′-UTR (Figure 1A). An ORF within the *OsCERK* gene encodes a polypeptide of 607 amino acid residues, with a predicted molecular mass of 67.29 KDa and a pI of 6.19.

Database searches indicated that proteins with homology to CERK are ubiquitous in multicellular organisms including vertebrates, invertebrates and plants. *OsCERK* clusters with the ceramide kinase of *Arabidopsis thaliana* and possible ceramide kinase orthologues in *Populus trichocarpa* and *Vitis vinifera* (Figure 1B). Moreover, putative CERKs clearly cluster into vertebrate, invertebrate and plant lineages, respectively. CLUSTAL W alignment of the amino acid sequences of OsCERK with putative CERKs from various organisms showed that the predicted OsCERK protein has a conserved CERK-specific motif CXXXCXXC that is critical for the enzymatic activity of hCERK [12] (Figure 1C), and five conserved domains (C1–C5) previously identified in human CERK (Figure S1). Gaps in the alignment between OsCERK (or ACD5) and hCERK due to sequence divergence may correspond to regions important for substrate recognition and regulatory mechanisms specific to plants and animals, respectively. A search using the SMART online bioinformatics tool revealed a diacyl glycerol kinase (DAGK) domain in residues 139–357 of OsCERK and in 164–366 of ACD5 as reported before [6].

Steady-state levels of *OsCERK* transcripts were highest in leaves, followed by the stems, with almost no expression in roots when compared with leaves (Figure 1D). Fresh rice leaf extracts harbored CERK enzyme activity, phosphorylating C6-NBD-ceramide to give C6-NBD-ceramide-1-phosphate, as did recombinant OsCERK and rACD5, respectively (Figure 1E, F). Almost no CERK activity was detected in the root extract (Figure 1E), which is consistent with the low levels of OsCERK transcripts (Figure 1D).

### Biochemical characterization of recombinant OsCERK

**Figure 2:**
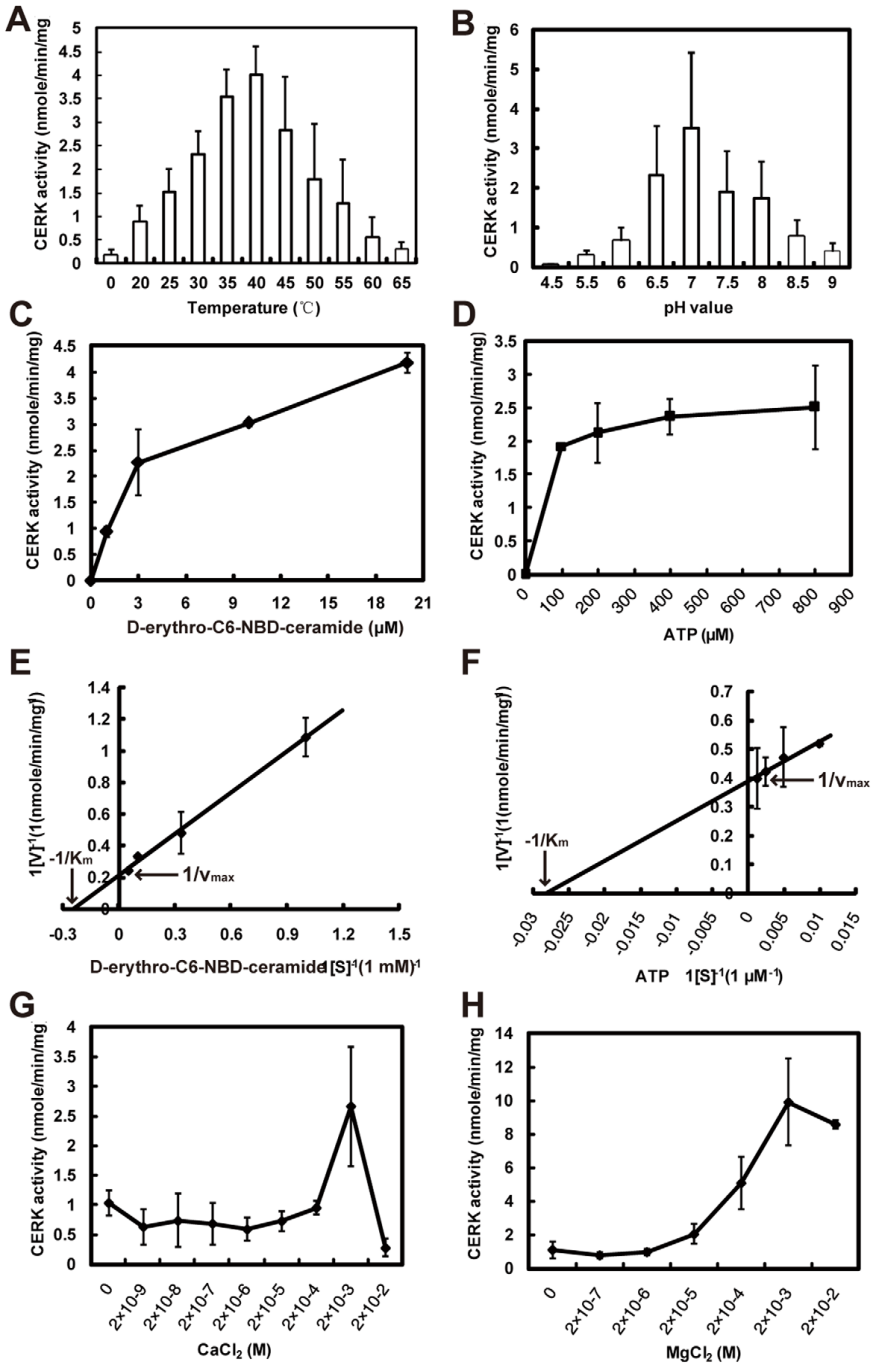
Biochemical characterization of the recombinant rice ceramide kinase. (A) Effect of different temperatures on CERK activity. (B) Effect of different pH values on CERK activity. (C) and (D) Michaelis-Menten representation for OsCERK activity with increasing concentrations of D-*erythro*-C6-NBD-ceramide and ATP, respectively. (E) and (F) Lineweaver-Burk plot of OsCERK activity. (G) Effect of calcium on ceramide kinase activity; for this experiment, ceramide phosphorylation was determined in the absence of MgCl_2_. (H) Effect of magnesium on ceramide kinase activity; for this experiment, the effect on activity by MgCl_2_ was measured in the absence of CaCl_2_. For (A)–(H), 1 µg purified recombinant OsCERK was used for OsCERK activity assay. The reaction time was 30 min. Values are means ± standard deviation (SD) of four replicates from two independent experiments.

Purified His-tagged OsCERK had an optimum reaction temperature of 40°C and an optimum pH value of 7, respectively (Figure 2A, B). With synthetic C6-NBD-ceramide as substrate, the reaction catalyzed by OsCERK showed classical Michaelis-Menten kinetics (Figure 2C, D), with an apparent K_m_ of 4.1 µM and an apparent V_max_ of 4.74 nmole^−1^ min^−1^ mg^−1^ protein for D-*erythro*-C6-ceramide (Figure 2E). The K_m_ and V_max_ for ATP was 35.36 µM and 2.58 nmole^−1^ min^−1^ mg^−1^ protein, respectively (Figure 2F). To identify the role of divalent ions for CERK activity, the effect of Ca^2+^ and Mg^2+^ on OsCERK activity was determined with C6-NBD-ceramide as substrate. In the absence of Mg^2+^, Ca^2+^ at <200 µM produced no change in CERK activity (Figure 2G), though the activity was increased at 2 mM. In the absence of Ca^2+^, the activity of CERK was increased by Mg^2+^ in a concentration-dependent manner (Figure 2H), which closely resembled the properties of hCERK [8]. Moreover, the addition of Ca^2+^ did not boost the reaction rate when 15 mM MgCl_2_ was present in the reaction system (data not shown). Thus, unlike ACD5 [6] and hCERK [8], which can be activated by Ca^2+^, OsCERK activity was mainly activated by Mg^2+^, and to a smaller extent by Ca^2+^.

### Complementation of ceramide kinase-deficient *acd5* mutant by OsCERK

**Figure 3:**
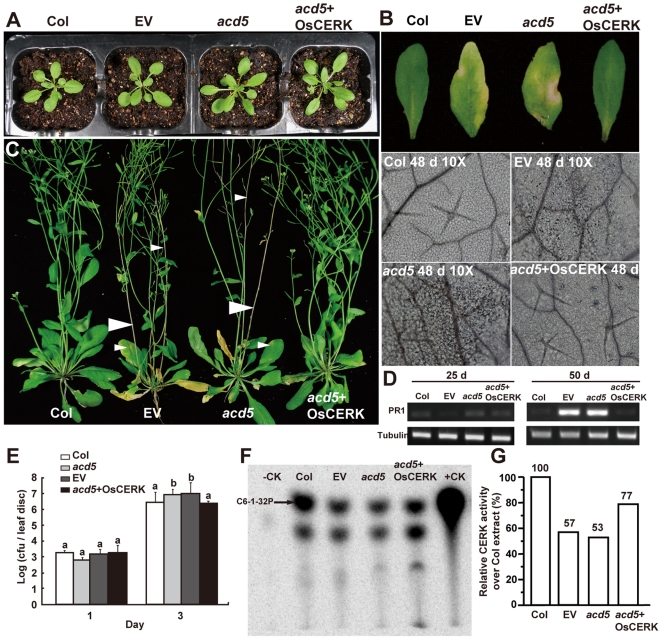
Complementation of *Arabidopsis acd5* mutant plants by *OsCERK*. (A) Comparison of early development between *acd5* mutant plants expressing *OsCERK* and nontransgenic plants. Note that no phenotype differences were observed in 25-day-old transgenic *acd5* plants expressing *OsCERK* (*acd5*+*OsCERK*) and empty vector when compared with wild-type and *acd5* plants. (B) Phenotypes of 48-day-old plant leaves (upper part) as shown in (A). Cell death was determined by trypan blue staining on the indicated days in leaf tissue (lower part). Note that *acd5* and transgenic *acd5* plants expressing EV showed obviously spontaneous cell death in both leaves and stained tissues; in contrast, *acd5* plants carrying *OsCERK* showed a completely normal phenotype, just as wild-type plants. This experiment was repeated twice with similar results. Each time 9–12 leaves from at least 6 plants were used for trypan blue staining and over 85% showed full complementation. (C) Phenotypes of 55-day-old plants as shown in (A). Arrowheads indicate the cell death phenotype of *acd5* and transformed plants carrying the empty vector in leaves and stems. Note the lack of cell death regions in the transformed *acd5* plants carrying OsCERK. At least ten independent lines were obtained. More than 100 plants were observed and phenotypic complementation was 95% in all homozygous lines. (D) Steady-state levels of *PR1* gene transcripts in wild-type and transgenic plants at different times. Tubulin indicates the loading control. This experiment was repeated twice with equivalent results. (E) Growth of *P. syringae* virulent strain DG3 in the indicated genotypes after infection at OD600 = 0.001. Lesion-free plants were inoculated with bacteria at 3 wk of age. The mean value of the growth of bacteria in six leaves is indicated in each case. Letters indicate that values are different using Student-Newman-Keuls test (*P*<0.05). Bars indicate standard deviations. This experiment was repeated three times with similar results. (F) CERK activity assay of plant extract in the indicated plants. Equal amounts of protein were extracted from 50-day-old leaves as described in Materials and Methods. Arrow indicates the formed product C6-1-^32^P. Purified ACD5 recombinant protein was used as the positive control (+CK). For negative control (−CK) no isotope-labeled ATP was added. This experiment was repeated twice with equivalent results. (G) Amount of C6-1-^32^P quantified from panel (F).

To assess the *in planta* function of OsCERK, we characterized T3 homozygous lines of the *Arabidopsis acd5* mutant harboring a cDNA of OsCERK. Several phenotypes of *acd5* were complemented by OsCERK, including spontaneous cell death and elevated *PR1* transcript levels late in development (Figure 3 A–D), and enhanced disease susceptibility to virulent *Pseudomonas syringae* strain DG3 of younger (21 day old) plants that lack cell death (Figure 3E). Thus, the *OsCERK* transgene complemented multiple phenotypes of *acd5*.

Importantly, in plant extracts, CERK activity was partially restored in *acd5*+*OsCERK* plants (Figure 3F, G). We noticed that an extra band appeared with all plant extracts that was more intense with *acd5*+*OsCERK* plant extracts. We speculate that OsCERK overexpression activates other lipid kinases and/or the C6 ceramide substrate was incorporated into larger lipids. Taken together, the *acd5* phenotypes were rescued by the *OsCERK* transgene.

### Effects of site-directed mutations in a conserved C-rich motif on OsCERK activity and function

**Figure 4:**
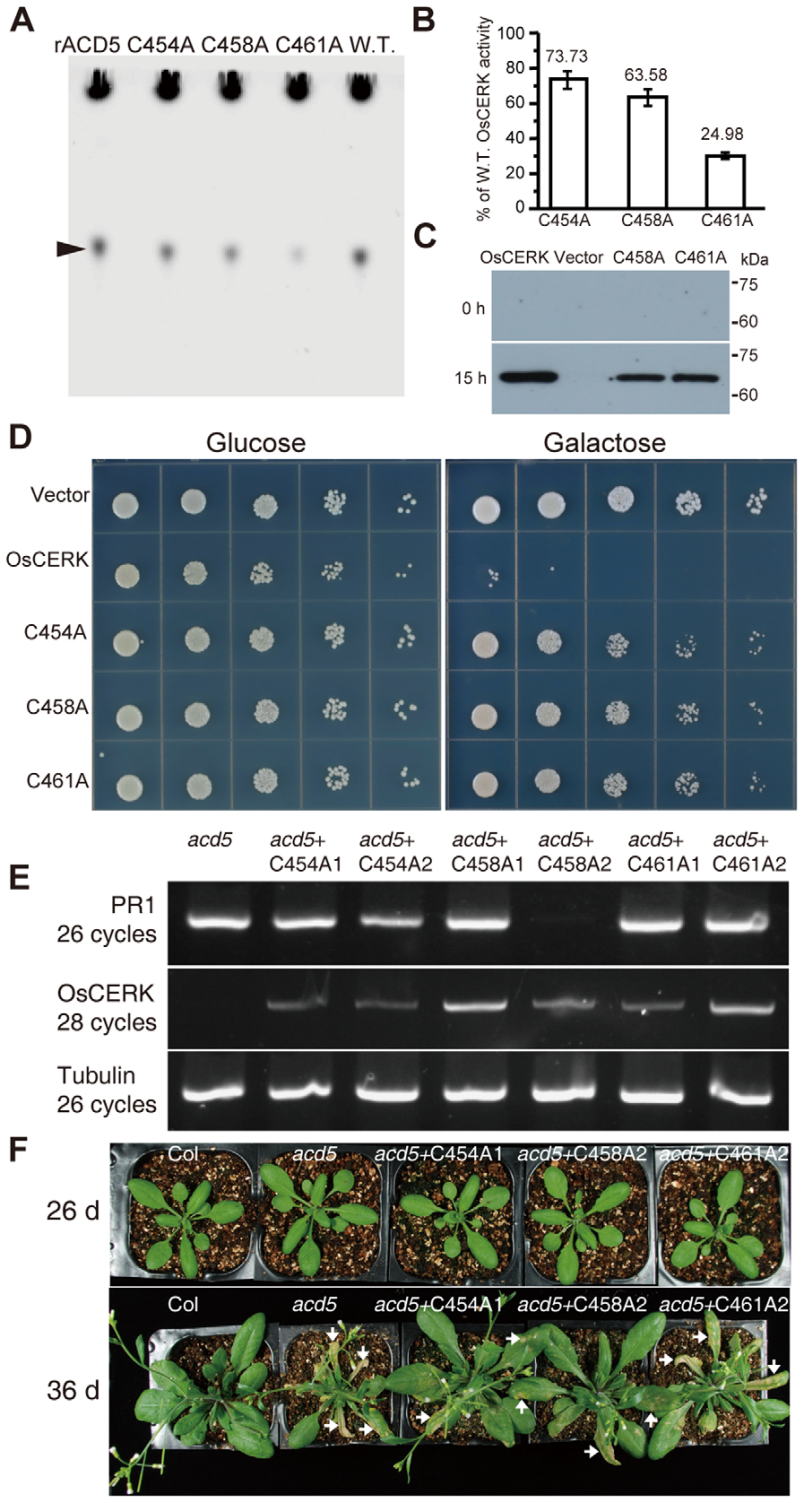
Effects of mutations in highly conserved motif on OsCERK activity and function. (A) Effects of mutations in the highly conserved CXXXCXXC motif on OsCERK activity. Site directed mutagenesis of OsCERK was performed as described in Materials and Methods. Equal quantities of protein were added into the 100 µl reaction system, and 1 µl of reaction mix was resolved on a TLC plate after 30 min incubation at 40°C. Purified recombinant ACD5 protein (rACD5) was used as a positive control. Arrowhead indicates the product of C6-NBD-Cer-1P. W.T. means recombinant OsCERK. (B) Relative OsCERK activity quantified from same samples as in panel (A). The values on the vertical axis, were normalized to wild-type (W.T.) rOsCERK activity at 100%. Note that the three variant proteins showed decreased activity, with the C461A variant showing the least activity. Bars represent the mean ± SD of two independent experiments. (C) Western blots with Flag antibody show the stable production of OsCERK and its modified variants in yeast. This experiment was repeated twice with equivalent results. (D) Effects of OsCERK and modified versions of OsCERK on yeast growth. Note the dramatic growth inhibition in yeast expressing OsCERK. This experiment was repeated four times with equivalent results. (E) Expression level of *OsCERK* and *PR1* in *acd5* and *acd5* transgenic plants. Two different lines in each site-mutated OsCERK were used. Note that the plant materials of line *acd5*+C458A2 were taken from 26-day-old seedlings as shown in (F), the other transgenic lines and *acd5* were taken from 36-day-old plants as shown in (F). (F) Comparison of phenotype between *acd5* mutant plants expressing mutated *OsCERK* and non-transgenic *acd5* plants. At least 50 plants from 2 independent lines were used for phenotype identification in each site-mutated OsCERK plants. Arrows indicate cell death lesions.

To discern the role of the CXXXCXXC motif conserved among all CERK species, we individually mutated each of the three cysteine residues in OsCERK, and assayed CERK activity using purified recombinant enzymes. The three mutant proteins displayed 25% (C461A), 64% (C458A) or 74% (C454A) residual activity, respectively (Figure 4A, B).

Because yeast lack a clear ceramide kinase homolog [8], it seemed possible this would be a good surrogate system to assess the effect of mutations on OsCERK function. Indeed, FLAG-tagged wild-type OsCERK expressed under galactose control dramatically suppressed yeast growth (Figure 4C, D), presumably due to a lipid imbalance conferred by OsCERK. In contrast, two mutated versions (C461A and C458A) still accumulated in yeast (Figure 4C), but had a reduced effect on growth (Figure 4D). We also attempted to assess the effects of the C454A variant on yeast growth, but the protein failed to accumulate. Based on the effects of mutation of C461 or C458, the conserved CXXXCXXC motif is important for OsCERK activity and impacts its function.

To test the consequences of disrupting the CXXXCXXC motif *in planta*, we transformed the site-directed OsCERK mutants into *acd5*. More than 5 independent lines for each site-directed mutation of OsCERK were obtained. At least 50 plants from each of 2 independent lines were used for phenotypic analysis of transgenic *acd5* harboring each site-mutated OsCERK. Lines with the same construct showed similar phenotypes; representative lines are shown in Figure 4. As shown in Figure 4E, the *OsCERK* gene was stably expressed in transgenic *acd5* plants; no *OsCERK* expression was detected in *acd5* control plants. Importantly, *acd5* plants transformed with *OsCERK* harboring mutations in the conserved motif still showed some spontaneous cell death and elevated *PR1* expression late in development (Figure 4F, 36 d). Similar to *acd5*, leaves taken from transgenic plants prior to spontaneous cell death showed no *PR1* expression (Figure 4E line *acd5*+C458A2).

Interestingly, the onset and extent of cell death in *acd5* carrying C461A OsCERK at 36 days and thereafter was similar to that seen with *acd5* plants. However, *acd5* carrying C454A or C458A OsCERK, respectively, showed less cell death than *acd5* at 36 days. Thus, the mutated versions of OsCERK showed varying degrees of defects in complementing the *acd5* cell death phenotype. Base on all above data, the highly conserved CXXXCXXC motif and particularly C461 is vital for OsCERK activity and function.

### Effects of CERK inhibitors on OsCERK activity and cell death

**Figure 5:**
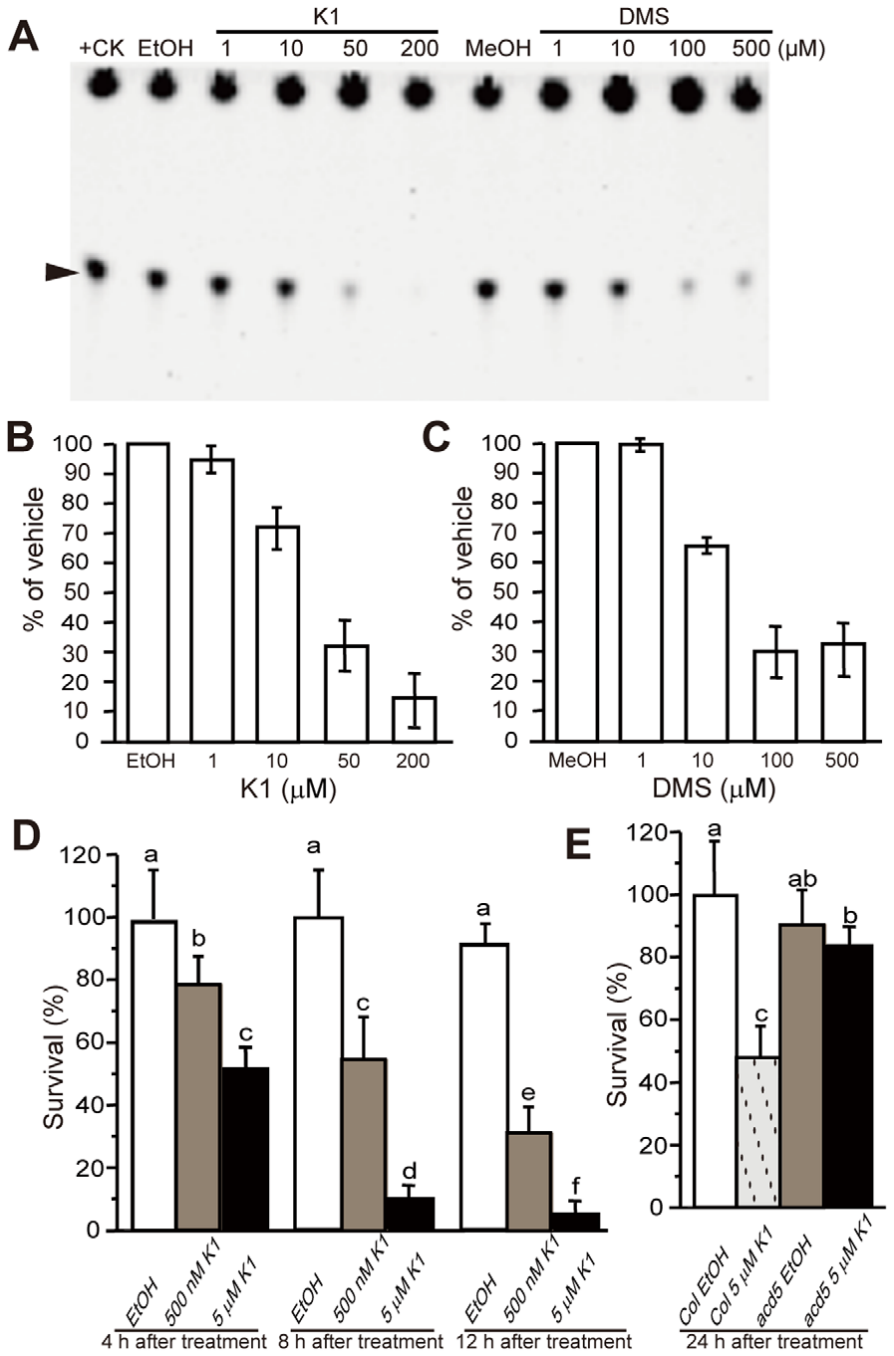
Inhibition assay and effects of site-mutation on OsCERK activity. (A) Effect of ceramide kinase inhibitors K1 and DMS on OsCERK activity. The activity of OsCERK was assayed as described in Materials and Methods using TLC and scanned for NBD fluorescence. Arrowhead indicates the formed product C6-NBD-Cer-1P. K1 and DMS were used at different concentrations. Purified OsCERK recombinant protein was used as a positive control (+CK). EtOH (0.5%) and MeOH (0.5%) indicated solvent controls for K1 and DMS, respectively. (B) and (C) Quantification of data from (A). Bars indicate means ± SD of two independent experiments. (D) Effect of the ceramide kinase inhibitor K1 on rice protoplasts. Protoplasts were isolated from 10-day-old rice seedling and treated with K1 (0.5 µM and 5 µM) for the indicated times under light. (E) Effect of ceramide kinase inhibitor K1 on *Arabidopsis* protoplasts. Protoplasts were isolated from 18-day-old wild-type and *acd5* mutant plants and treated with 5 µM K1 for 24 h under light. The percentage of surviving cells in (D, E) was estimated by FDA staining. These experiments were repeated three times with similar results. Letters indicate that values differed in Fisher's protected least significant difference (PLSD) test, a post hoc multiple *t* test (*P*<0.05). Error bars indicate standard deviations. Control treatment was with 0.15% ethanol (the solvent in which K1 was dissolved).

In order to discern whether OsCERK activity is required for cell viability, we investigated the effect of reducing OsCERK activity with recently commercialized CERK inhibitors, such as N,N-dimethylsphingosine (DMS), which partially inhibits hCERK activity [8], and the ceramide kinase inhibitor K1 (K1), a specific noncompetitive CERK inhibitor [33]. As shown in Figure 5, OsCERK activity was inhibited by K1 and DMS in a concentration dependent manner, and K1 showed stronger inhibition than DMS (Figure 5A, B, C), which is consistent with the inhibitory effect on hCERK. OsCERK activity decreased by about 70% in 100 µM DMS and 50 µM K1. K1 caused greater than 90% inhibition of OsCERK activity at 200 µM concentration, demonstrating that K1 can almost completely abolish OsCERK activity.

To examine the effect of K1 on cell viability, rice and *Arabidopsis* protoplasts were treated with K1. In rice protoplasts treated with different concentrations of K1, cell death occurred in a dose- and time-dependent manner (Figure 5D). In *Arabidopsis* protoplasts, K1 induced cell death much less in *acd5* than in wild-type plants (Figure 5E). These data are consistent with K1 specifically affecting CERK activity, which is crucial for the maintenance of cell viability.

### Characterization of ceramide-induced PCD in rice

To assess whether ceramides, the substrates of OsCERK, can induce cell death in rice, we isolated rice protoplasts and treated them with ceramides. We used short-chain ceramides because of the difficulty in ensuring that longer chain ceramides efficiently enter plant cells. In animal cells, short chain ceramides are converted into longer ceramides *in vivo* [34]; it seems likely that similar events occur in plants. As shown in Figure S2, both C2- and C6-ceramides induced cell death in a time-dependent manner, whereas C2- and C6-dihydroceramide, the biosynthetic precursors of ceramides, had almost no effect at the same concentration as ceramides, similar to what has been reported in animal and *Arabidopsis* cells [2], [6]. C6-ceramide and C2 similarly induced rice protoplast cell death. However, the concentration of C6 that induced cell death was 2 times higher than that of C2 (Figure S2A, B), suggesting that it is more difficult for C6 than C2 to enter protoplasts. Cell death appeared to be programmed, since 12 h after treatment with C6-ceramide, a clear DNA ladder was seen (Figure S2D) and nuclear fragmentation characteristic of apoptotic cells was seen by TUNEL staining (Figure S2C) [32], [35].

**Figure 6:**
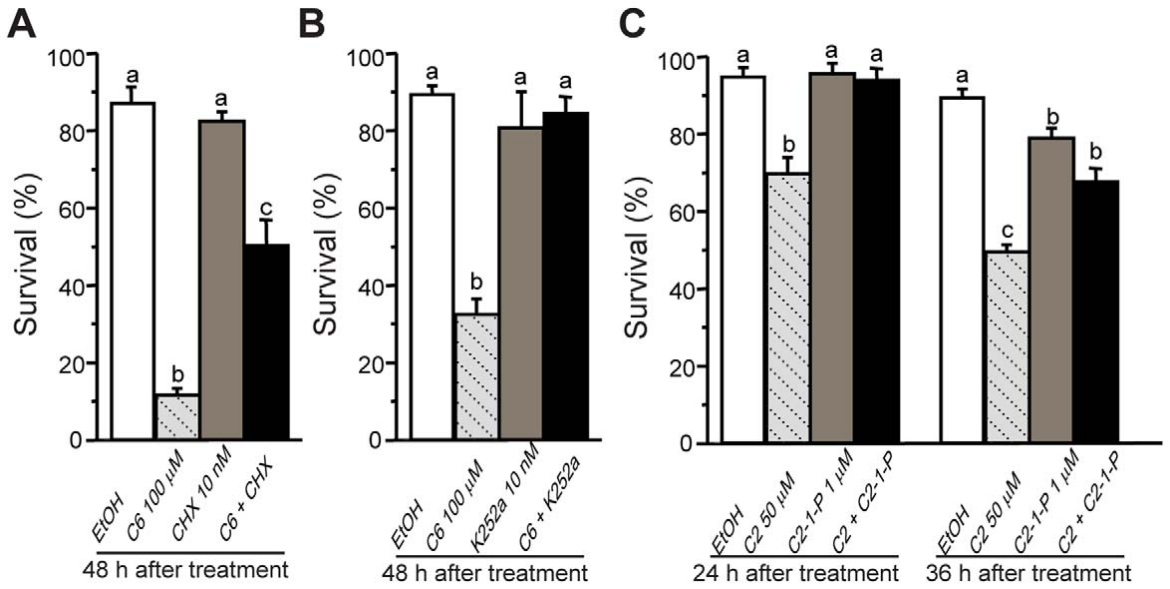
Effects of ceramide related inhibitors on cell death induction. (A) Effects of cycloheximide (CHX) on C6 ceramide-induced cell death. (B) Effects of K252a on C6 ceramide-induced cell death. (C) Effects of C2 ceramide-1-phosphate on C2 ceramide induced cell death. Protoplasts were treated with less than 0.15% solvent or the indicated reagent. Percentage of surviving cells was estimated by FDA staining. These assays were repeated three times with similar results. Letters indicate that values are different using Fisher's PLSD test (*P*<0.05). Bars show standard deviations.

Cell death induced by C6 ceramide in rice involved new protein synthesis and protein phosphorylation. This was evidenced by the inhibition of each process using cycloheximide or the serine/threonine kinase inhibitor K252a, respectively, which suppressed cell death to varying extents (Figure 6A, B). Treatment with the inhibitors alone at the concentrations used did not affect viability of protoplasts. Additionally, as was found in *Arabidopsis*, the presence of C2-1-phosphate partially blocked cell death induced by C2 ceramide in rice (Figure 6C), supporting the idea that the balance between ceramides and their phosphorylated derivatives modulates cell death in plants.

**Figure 7:**
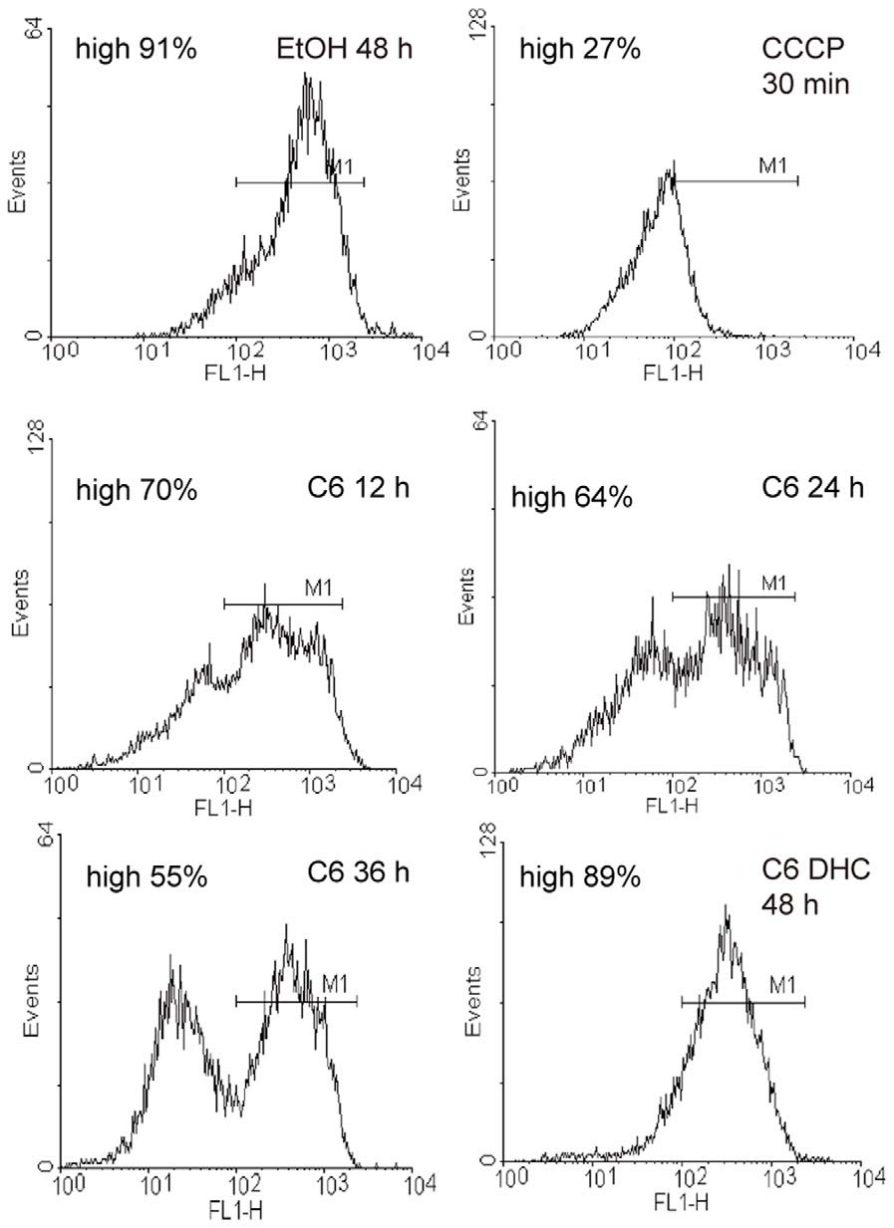
Ceramide induced programmed cell death is associated with Δψ_m_ loss in rice protoplasts. Mitochondrial membrane potential Δψ_m_ loss detected by flow cytometry. Protoplasts were cultured under light for the indicated times with 0.1% ethanol (solvent control), 100 µM C6, 100 µM C6-DHC and 100 µM protonophore CCCP (positive control), respectively. Δψ_m_ was analyzed by flow cytometry using the fluorescent probe DiOC_6_(3). Results are from a single analysis, representative of three independent experiments that showed similar results. M1, region of high Δψ_m_. The percentages in each panel indicate the proportion of the cells with high Δψ_m_.

As in *Arabidopsis*, rice protoplasts showed functional changes in mitochondria after ceramide treatment. C6 ceramide, but not C6-DHC, induced a time-dependent loss in mitochondrial membrane potential (Δψ_m_), as assessed by flow cytometry using DiOC_6_(3) staining (Figure 7). Protoplasts treated with carbonyl cyanide m-chlorophenylhydrazone (CCCP), as a positive control, showed dramatic Δψ_m_ loss after 30 min treatment. The results of treatment with C2 were similar to C6 (data not shown). Although Δψ_m_ loss was seen in rice and *Arabidopsis* after ceramide treatment [32], the timing of Δψ_m_ loss in rice did not precede cell death onset as was previously observed in *Arabidopsis* [32]. Thus, there may be differences in the relative importance of Δψ_m_ loss in rice and *Arabidopsis*.

## Discussion

We showed here that OsCERK both biochemically and genetically functions as a *bona fide* ceramide kinase that is important for plant cell survival and that ceramides modulated by OsCERK can induce programmed cell death in rice. Rice ceramide kinase requires a CERK-specific CXXXCXXC motif for its function *in planta* and its enzymatic activity *in vitro*. This suggests an evolutionarily conserved and key function for the CXXXCXXC motif in CERK enzymes.

### Novel biochemical properties of OsCERK

OsCERK activity is stimulated by Mg^2+^, but only modestly Ca^2+^. This contrasts with the properties of human CERK, which shows Ca^2+^ dependence and is stimulated by Mg^2+^ in the micromolar concentration range [8]. The differences in the biochemical properties of the human and rice enzymes might be linked to the domains present in hCERK versus OsCERK and/or their respective structures. hCERK contains a calcium/calmodulin binding motif of the 1-8-14 type B [8] that may modulate calcium/calmodulin binding to CERKs and control their function; this motif is absent from the plant enzymes. Stimulation of CERK activity in plants may need a higher concentration calcium than the human enzyme; indeed, ACD5 activity was significantly stimulated by 100 mM Ca^2+^ [6]. OsCERK was most active in the neutral pH range from 6.5 to 7.5, with optimal activity at pH 7.0. This pH dependence is quite similar to hCERK and is different from ACD5 (optimal pH 8.2) [6]. The K_m_ with D-*erythro*-C6-NBD-ceramide was 4.1 µM, much lower than the previous reported K_m_ values for CERK from other organisms. The K_m_ for ATP, 36.35 µM was similar to hCERK. The activity may vary with substrate, substrate delivery [36], reaction system and detection methods of activity. Notably, OsCERK has a temperature optimum of 40°C, higher than ACD5 or hCERK. This high temperature optimum may be related to fact that rice can grow at higher temperatures.

### The CXXXCXXC motif is important for OsCERK activity and function

The highly conserved CXXXCXXC motif of CERK, between the C4 and C5 domains, is essential for hCERK enzyme activity and cellular localization [12]. However, the role of the cysteine motif for the full *in vivo* function(s) of hCERK has not been reported. Our site-directed mutagenesis results indicate that each of the three cysteines contributes to the enzyme activity of OsCERK, with the mutation C461A that removes the third cysteine showing the strongest effect. *In planta*, each of the three cysteines tested were important, since none of the site-directed mutants could fully rescue of the cell death phenotype of *acd5* plants. In the surrogate yeast system, we also confirmed that C458A and C461A did not affect yeast growth the way the wild-type protein did. Thus, this conserved C-rich motif is also important for the *in planta* function of OsCERK.

In hCERK, the first cysteine in the motif is most important for its enzymatic activity [12], whereas in OsCERK, mutation of the third cysteine had the most dramatic effect on activity and *in planta* function. The reason for this difference is not clear. Whether the motif also impacts the localization of OsCERK in plants awaits further determination. Interestingly, an identical cysteine motif has been characterized in the radical S-adenosylmethionine super family of enzymes, where the motif provides a metal binding site that is essential for activity [37]. Whether CERK is also regulated by metal coordination will require further research.

### Ceramides affect cell viability and Δψ_m_ in rice

CERK phosphorylates ceramide to yield C1P, a molecule with interesting signaling properties [38]. Ceramides, as the substrates of CERK, are closely involved in PCD, and affect many cellular events in animals [2]. Ceramide also activates programmed cell death in *Arabidopsis* [6] and rice. As was observed in *Arabidopsis* [6], ceramide-induced cell death in rice is also suppressed by the presence of C1P. This suppressive effect could result from direct competition of C1P with ceramide in affecting the same cellular target or through modulation by ceramide phosphate of signaling. We favor the latter hypothesis, since protein phosphorylation, a modification often associated with signal transduction, is important for ceramide-induced cell death in rice and C1P has signaling roles in animals.

In *Arabidopsis*, a mitochondrial permeability transition, just like in animal apoptosis, is one of the early events in PCD induced by C2-ceramide [32]. In rice protoplasts Δψ_m_ loss was also observed during PCD induced by short-chain ceramides, however, the membrane potential loss appears simultaneously with PCD, suggesting that in rice Δψ_m_ loss may not be essential for PCD induced by ceramide.

In conclusion, a ceramide kinase from rice was cloned and characterized. The ceramide kinase inhibitors, which were characterized on CERK from animals, almost completely blocked OsCERK activity and induced cell death in both rice and *Arabidopsis*. By site- directed mutagenesis of the conserved CXXXCXXC motif in organisms, three highly conserved cysteines were shown to be vital for CERK activity and function. Since CERK is one of the key enzymes in sphingolipid metabolism, our data offers important information for understanding the conserved sphingolipid functions in animals and plants. It will be interesting to pursue the further role of CERK in plant developmental processes during environmental stress.

## Materials and Methods

### Plant growth and plant infections

Rice (*Oryza sativa* ssp. *japonica* cv. Nipponbare) seeds were planted in soil after two days of soaking in water and grown in a climate incubator under the following conditions: photo flux density, 225 µmol m^−2^ s^−1^, 16 h light/8 h dark, with a day temperature of 28°C under 80% relative humidity and a night temperature of 26°C under 60% relative humidity. *Arabidopsis thaliana* plants (ecotype Columbia) were grown in a 16 h light/8 h dark cycle at 22°C under 60% relative humidity. *Pseudomonas syringae* pv. *maculicola* strain DG3 (virulent) was inoculated as described previously [31], [39].

### Full length cDNA cloning and RT-PCR analysis

Total RNA was isolated from 2-week-old seedlings using Trizol Reagent according to the manufacturer's instructions (Invitrogen, Carlsbad, CA, USA). About 1 µg total RNA was used for cDNA synthesis using RevertAid™ First Strand cDNA Synthesis Kit (Fermentas, EU). Aliquots (1 µl) of the product were used for PCR amplification using Pfu high-fidelity DNA polymerase (Tiangen, Beijing, China), with the forward primer 5′-ATGGAAGGCGGCGGCGAGGCGCTCT-3′ and reverse primer 5′ -CTACATGCCTGCTCTTTCCGGTGGC-3′. Cloning of the 5′- and 3′-UTRs was accomplished using a SMART RACE cDNA amplification kit (Clontech, Mountain View, CA, USA) according to manufacturer's instructions with the following gene specific primers: for 3′RACE: NGSP 5′-ATGGGTCCTGCGCGATATGACTT-3′, GSP 5′- GTTTCACCAACTGTGCGCTATGCTG-3′; for 5′RACE: GSP 5′-GATGACCAACATGAAGAACTCACTT-3′, NGSP 5′-ACCTGGTGAAGGGGCTGGAAGGA-3′. The full-length coding sequences were cloned into the pGEM-T vector (Tiangen) before sequencing (Invitrogen). The 5′- and 3′-UTRs were cloned into the pCR2.1 vector (Invitrogen) before sequencing.

For RT-PCR analysis, total RNA samples and Reverse-Transcription were done as described above. The cDNA contents in the different reverse transcription mixtures were normalized by amplifying the transcripts of β-actin using the primer 5′-CCATTGGTGCTGAGCGTT-3′ and 5′-CCCGCAGCTTCCATTCCTA-3′ in rice. Evaluation of the transcript levels of *OsCERK* in the different tissue was carried out using the normalized cDNA mixtures and the primer 5′-TGGCTCAACGGATGCTATT-3′ and 5′-ACGACCCTTGGACCAGACC-3′. For *Arabidopsis* expression analysis, the primer 5′-GTCACTTGCTGCCTTCGTTT-3′ and 5′-CTTCTTTCGTGCTCATTTTGC-3′ were for tubulin amplification as an internal control, the *PR1* expression was amplified using the primer 5′-GTGCTCTTGTTCTTCCCTCG-3′ and 5′-AGCCTTCTCGCTAACCCACA-3′.

### Mutagenesis of OsCERK

Site directed mutagenesis of *OsCERK* was performed using the following primers with Fast mutagenesis system (Trans, Beijing, China): C454A (5′-GACATCGGAAAACCATCTGCCGAACAAATTG-3′,5′-GCGATGGTTTTCCGATGTCTATTTTGATGGTA-3′), C458A (5′-CCATCTGCCGAACAAATGCTTTAATATGCAA-3′, 5′-GCATTTGTTCGGCAGATGGTTTTCCGATGTC-3′), C461A (5′- GAACAAATTGTTTAATAGCCAAAGGAACTTC-3′,5′-GCTATTAAACAATTTGTTCGGCAGATGGTTT-3′). The construct of pET28b: *OsCERK* was used as template for PCR amplification. All constructs were transferred to BL21 (DE3) and expressed using the conditions described below.

### Plasmid construction of yeast expression, spot assay and western blots

The wild-type and site-mutated coding regions of OsCERK were introduced into *Sal* I and *Xba* I digested pYES2+Flag expression vector (reconstructed from pYES2 vector). In the spot assay, yeast cells grown in SC-glucose medium for 1 day were adjusted to A600 of 0.1. Five microliters of five-fold serial dilutions of each yeast culture was spotted on to SC-glucose or SC-galactose medium and incubated for 2 days (glucose medium) or 3 days (galactose medium) at 30°C. For western blots, yeast cells cultured in liquid galactose medium were collected by centrifugation at 0 h and 15 h, respectively. Cell lysates were prepared using acid-washed glass beads (Sigma-Aldrich, St. Louis, MO, USA). Protein concentration was determined with a Protein assay kit (Bio-Rad) using IgG as the standard. Proteins (10–20 µg) were separated by SDS-PAGE (10%, v/v). Separated proteins were electrophoretically transferred to a nitrocellulose filter membrane (0.45 µm, Bio-Rad). Nonspecific binding was blocked with 5% (w/v) skim milk in TBST buffer (25 mM Tris-HCl, pH 7.4, 140 mM NaCl, 0.1% (w/v) Tween 20) for 1 h at room temperature. N-terminal Flag- fused proteins were detected using anti-Flag antibody (1∶2000 dilutions, Sigma-Aldrich, St. Louis, MO, USA) and developed using the enhanced chemiluminescence kit (ECL, Rockford, Thermo Scientific). The chemiluminescence was finally detected with photographic film (Kodak).

### Phylogenetic analysis

Based on analysis of sequence alignment described in Methods S1, the phylogenetic tree and executed bootstrap test were calculated and decided by the Neighbor-Joining method from MEGA 4.0 [40].

### Expression of OsCERK and ACD5 in *Escherichia coli* and purification of recombinant protein

The full-length *OsCERK* coding sequence was subcloned into the *E. coli* expression vector pET28b (Novagen) and transformed into BL21 (DE3) with the forward primer 5′- CGGGATCCGATGGAAGGCGGCGGCGAGGCGCTCT-3′ (*Bam*H I site) and reverse primer 5′ -TCACTCGAGCTACACCTCCGGTCCTGACGCAAAG-3′ (*Xho* I site). The transformed *E.coli* cultures were grown in LB medium for large-scale expression. Cells were grown at 37°C with shaking in LB medium. When the turbidity (A600) reached 0.5, IPTG (Merck) was added to 0.5 mM and growth was continued at 16°C overnight, then the cultures were harvested at 4°C. Lysates were analyzed by Western blots using anti-His tag antibody (Tiangen, Beijing, China). His-CERK was purified on Ni-NTA His-Bind resin (Novagen, USA) from 100 ml bacterial cultures, as described in the product manual. After purification, protein concentration was assayed with Easy Protein Quantitative Kit (Trans, Beijing, China). Purified enzyme was stored at −20°C after the addition of sterile glycerol to 50% (V/V). For ACD5, the coding region of *acd5* was introduced into pET28b (Novagen) with forward primer 5′-CGGGATCCGATGGAGGAAGGTCGTGACGACGAGT-3′ (*Bam*H I site) and reverse primer 5′ -TCACTCGAGTTATATCTCTGGACCAGATGCGAAC-3′ (*Xho* I site), and the expression system and conditions was same as OsCERK.

### OsCERK activity assay and biochemical characterizations

CERK activity was measured as previously described with modification [41]. Briefly, mixed micelles containing 1.25 mM (0.125%) Triton X-100, 0.2 mM cardiolipin (Sigma-Aldrich, St Louis, USA) and 50 µM D-*erythro*-C6-NBD-ceramide (Avanti Polar lipids, Alabaster, AL, USA), were prepared by drying lipids from ethanol stocks and then sonicating in reaction buffer [20 mM Hepes (pH 7.4), 10 mM KCl, 15 mM MgCl_2_ and 1 mM dithiothreitol (DTT)]. The micelles were diluted fivefold with reaction buffer, and 1 mM ATP (Amresco) was added. The 100 µl reactions were started with the addition of 1.0 µg His-CERK and reacted at 40°C for 30 min in the dark, and then transferred to ice. The reaction mixture (1 µl) was spotted directly onto silica TLC plates (Gel60 F254: Merck, Darmstadt, Germany). The products were separated from the substrate by developing the plate in a solvent system comprising butanol/acetic acid/water (3∶1∶1, by volume). Fluorescence was detected with Typhoon Trio^+^ scanner (GE Healthcare) in blue fluorescence mode. The C6-NBD-ceramide-1-phosphate products were identified by comparison with C6-NBD-ceramide (Avanti Polar lipids, Alabaster, AL, USA) and quantified using IQTL software (GE Healthcare). The specific activity was determined using standard curves with known concentrations of D-*erythro*-C6-NBD-ceramide. To test the effect of pH on the reaction rates, a series of reaction buffers containing 10 mM KCl, 15 mM MgCl_2_ and 1 mM dithiothreitol were prepared with 50 mM citrate (pH 4.5, 5.5 or 6.0), 50 mM Hepes-HCl (pH 6.5 or 7.0) or 50 mM Tris-HCl (pH 7.5, 8.0, 8.5 or 9.0). The K_m_ value was determined using the Lineweaver-Burk plot method.

For OsCERK activity of plant extracts, crude plant extracts were prepared as described previously [6]. The protein concentration was determined with Easy Protein Quantitative Kit (Trans, Beijing, China). CERK activity of rice lysates using C6-NBD-ceramide as a substrate was done with crude lysates using isotope-labeling method with modification [42]. Briefly, 50 µl of leaf lysates (60 to 120 µg of protein) in a total volume of 100 µl was reacted at 40°C for 30 min in the dark and terminated by adding 600 µl chloroform∶methonal (1∶1) and 265 µl of 1 M KCl in 20 mM MOPS buffer (pH 7.2). The reaction mix was analyzed by TLC developed with H_2_O∶acetic acid∶methanol∶acetone∶chloroform (5∶10∶15∶20∶50). The TLC plates were autoradiographed for 1–2 h in a phosphor imager cassette and scanned on a STORM 860 (GE Healthcare).

### Molecular complementation of *acd5* mutant

The full length *OsCERK* coding sequence was subcloned into pRT104 vector [43] with the forward primer 5′-TCACTCGAGATGGAAGGCGGCGGCGAGGCGCTCT-3′ (*Xho* I site) and the reverse primer 5′-CGGGATCCCTACACCTCCGGTCCTGACGCAAAG-3′ (*Bam*H I site). The plasmid pRT104∶*OsCERK* was digested with *Pst* I, and cloned into the same sites of pCAMBIA1305.1 vector (CAMBIA). For *OsCERK* mutant constructs (C454A, C458A, C461A), the mutated constructs for prokaryotic expression were cut with *Bam*H I and *Xho* I, and the site-mutated OsERK regions were introduced into *Bam*H I and *Sal* I digested pCAMBIA1300′ vector (the 35S promoter and NOS terminal were introduced into pCAMBIA1300). The obtained wild-type and mutant OsCERK constructs were transformed into *Agrobacterium tumefaciens* strain EHA105, which was used for transformation of *acd5* mutant plants by the floral dip method [44]. The transformed progeny were selected using 1/2 MS plates (solidified with 0.8% tissue culture agar) supplemented with 25 µg/ml hygromycin (Newprobe, Beijing, China). After selfing the T0 plants, lines homozygous for the transgene were chosen for further study.

### Visualization of dead cells

Fresh tissue was boiled in lactophenol (10 ml of lactic acid, 10 ml of glycerol, 10 ml of liquid phenol, and 10 ml H_2_O) containing 10 mg trypan blue for 30 S and boiled in 95% ethanol∶lacophenol (2∶1) for 1 min, and then cleared in 50% ethanol. Photographs were taken with a fluorescence microscope (Axio Imager A1, Carl Zeiss).

### Protoplast isolation and treatments

Rice protoplasts were isolated from 10-day-old seedling tissues as described previously with modifications [45]. Detailed method of this experiment is described in Methods S1. Protoplasts were isolated from leaves of 18-day-old *Arabidopsis* wild-type and *acd5* plants as described previously [6], [46].

*N*-acetyl-D-erythro-sphingosine (C2-ceramide), *N*-hexanoyl-D-sphingosine (C6-ceramide), N-acetyl-D-erythro-dihydrosphingosine (C2-dihydroceramide) and N-hexanoyl-D-erythro-dihydrosphingosine (C6-dihydroceramide) were purchased from Matreya (Pleasant GAP, PA, USA), and dissolved in ethanol. C2 ceramide-1-phosphate (C2-1-P) and N, N-dimethylsphingosine (DMS) were from Avanti Polar Lipids (Alabaster, AL, USA), and dissolved in ethanol. Cycloheximide (CHX: Sigma) and K252a (Enzo life science), were dissolved in DMSO (Sigma). Ceramide kinase inhibitor K1 (Calbiochem, LaJolla, CA, USA) was dissolved in ethanol. During treatments, the final solvent concentration was never more than 0.15%.

Statistical analyses were performed using Statview statistical package 5.0.1 (SAS Institute, Cary, North Carolina, USA) for Macintosh. Fisher's protected least significant difference (PLSD), a post hoc multiple t-test, was used. Results were considered significant when *P*<0.05. Data are presented as means ± SD (standard deviation). All experiments were repeated at least three times.

### Measurement of the mitochondrial membrane potential (Δψ_m_) by flow cytometry

To measure Δψ_m_, the mitochondrial probe 3,3-Dihexyloxacarbocyanine iodide [DiOC_6_(3), Molecular Probes, Eugene Oregon, USA] was used as described by [32]. Briefly, 5×10^5^ protoplasts were incubated with DiOC_6_ (3) (5 nM) for 10 min in culture medium in the dark, at room temperature. As a control for Δψ_m_ loss, protoplasts were incubated 100 µM protonophore carbonyl cyanide m-chlorophenylhydrazone (CCCP, Sigma-Aldrich, ST. Louis, USA) for indicated times before flow analysis. The protoplasts were washed once with W5 medium, collected by centrifugation at 800 rpm/min for 3 min. Dyed protoplasts were then stored in the dark for analysis by flow cytometry (BD FACS Calibur) using excitation with a single 488 nm argon laser. At least 30000 events were collected per sample. Data acquisition and analysis were performed using the CellQuest software (BD FACS Calibur, Becton Dickinson) and Win MDI2.9 (Scripps Research Institute, San Diego, CA).

### Accession numbers

The GenBank accession number for the nucleotide sequence of *OsCERK* is FJ765452. Other sequence data of the analyzed CERK proteins in this article can be found in the GenBank data library described in Methods S1.

## Supporting Information

Figure S1 **Sequence analysis of rice ceramide kinase (** ***OsCERK*** **).** The alignment of amino acid sequences of putative CERKs was executed by using CLUSTAL W. The consensus amino acids are shaded with a 60% identity threshold, amino acids are denoted as *colon* with an 80% threshold, evolutionarily conserved amino acids are denoted with an *asterisk*. The conserved domains (C1 to C5, [8]) in SPKs (sphingosine kinase) are indicated by *lines*. The highly conserved CXXXCXXC motif that is essential for CERK function, is indicated by shaded rectangle. The DAGK catalytic domain depicted by the SMART search tool is indicated by *shaded bars*.
(TIF)

Figure S2 **Ceramides induce programmed cell death in rice protoplasts.** (A) C2 ceramide-induced cell death in rice protoplasts for indicated times. Rice protoplasts were treated with 50 µM C2-ceramide (C2-cer) or C2-dihydroceramide (C2-DHC) under light. (B) C6 ceramide-induced cell death in rice protoplasts for indicated times. Rice protoplasts were treated with 100 µM C6-ceramide (C6-cer) or C6-dihydroceramide (C6-DHC) under light. The viability was determined by FDA staining. Standard errors in (A) and (B) are shown (*n* = 3). Letters indicate that values of viability differed in Fisher's PLSD test, a post hoc multiple *t* test (*P*<0.05). Control treatment was with 0.15% ethanol (the solvent for C2 or C6). (C) *In situ* detection of DNA fragmentation by TUNEL after C6-ceramide treatment. Rice protoplasts were treated with 0.15% ethanol and 100 µM C6-ceramide for 12 h and stained using the TUNEL method as described in Materials and Methods. The green signal (arrow) indicates a TUNEL-positive nucleus. Protoplasts were stained for nuclei using DAPI. Scale bars represent 10 µM. (D) The DNA ladder induced by 100 µM C6-ceramide after 12 h treatment.
(TIF)

Methods S1 **Includes appendixes.**
(DOC)
