# Supplementary figures and images for: A Conserved Cysteine Motif Is Critical for Rice Ceramide Kinase Activity and Function
Source: PLoS One. 2011 Mar 31;6(3):e18079. doi: 10.1371/journal.pone.0018079 (PMC3069040; doi:10.1371/journal.pone.0018079)

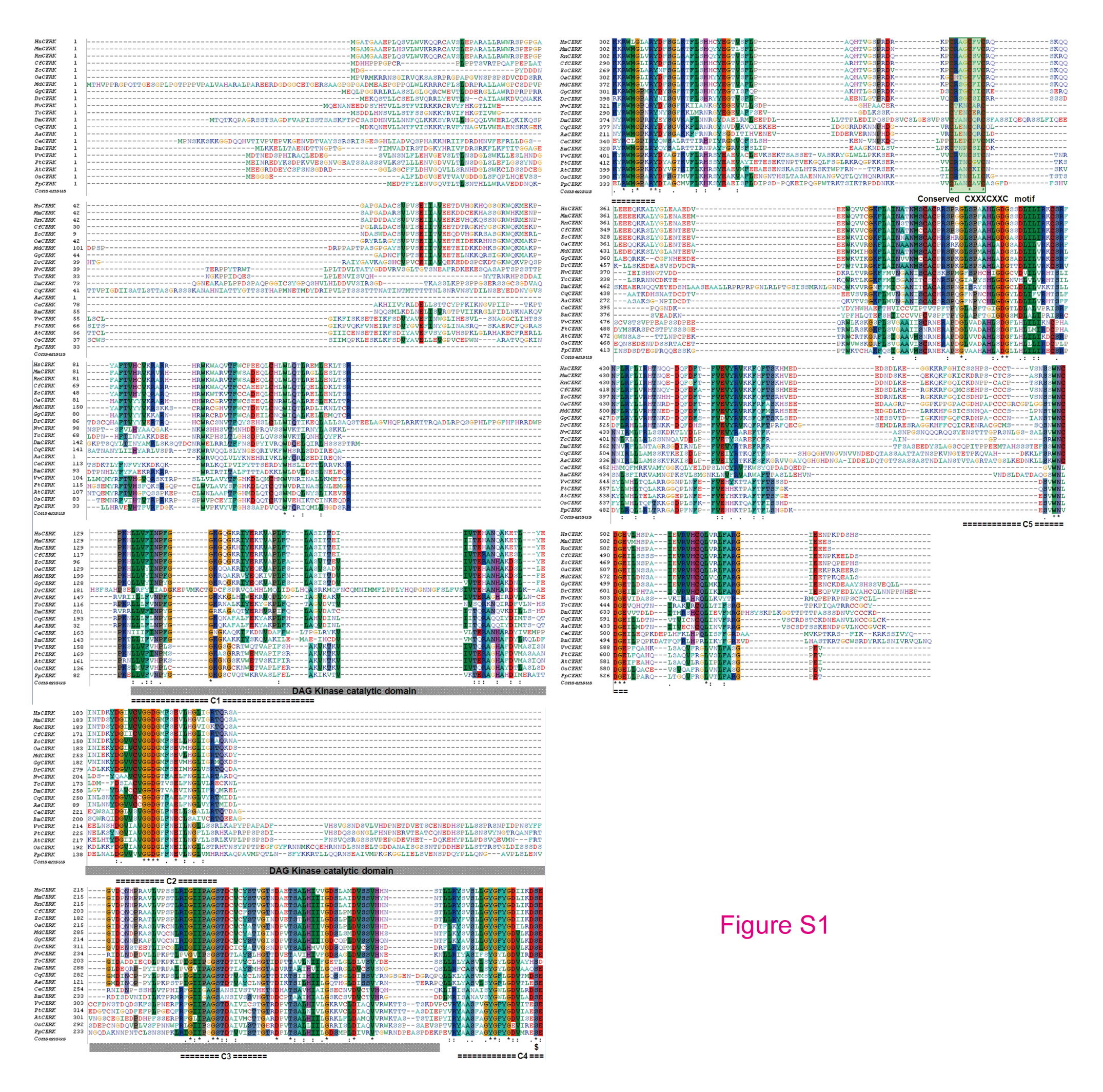

Supplement: Figure S1 — Sequence analysis of rice ceramide kinase ( OsCERK ). The alignment of amino acid sequences of putative CERKs was executed by using CLUSTAL W. The consensus amino acids are shaded with a 60% identity threshold, amino acids are denoted as colon with an 80% threshold, evolutionarily conserved amino acids are denoted with an asterisk. The conserved domains (C1 to C5, [8]) in SPKs (sphingosine kinase) are indicated by lines. The highly conserved CXXXCXXC motif that is essential for CERK function, is indicated by shaded rectangle. The DAGK catalytic domain depicted by the SMART search tool is indicated by shaded bars. (TIF) [file pone.0018079.s001.tif]

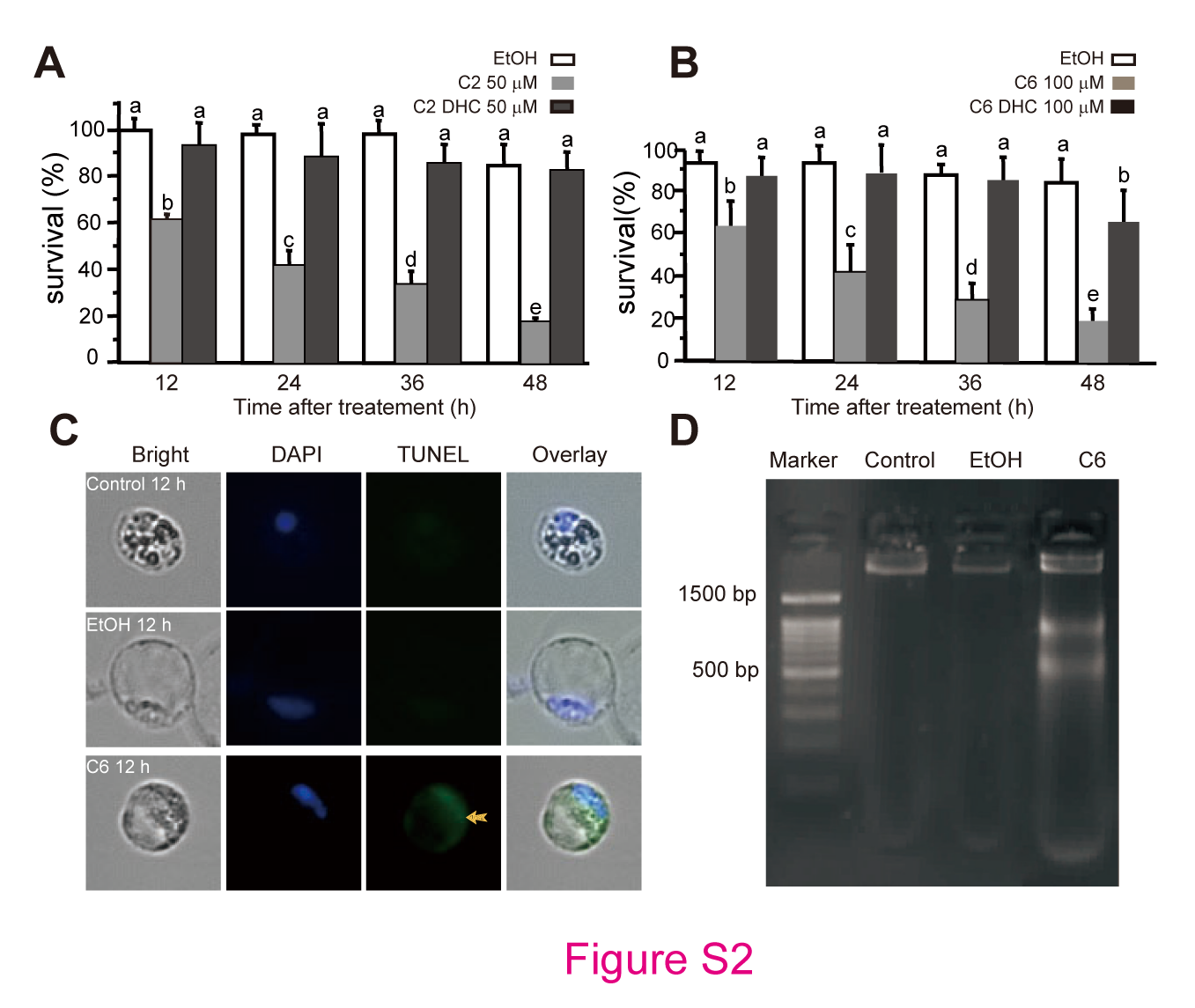

Supplement: Figure S2 — Ceramides induce programmed cell death in rice protoplasts. (A) C2 ceramide-induced cell death in rice protoplasts for indicated times. Rice protoplasts were treated with 50 µM C2-ceramide (C2-cer) or C2-dihydroceramide (C2-DHC) under light. (B) C6 ceramide-induced cell death in rice protoplasts for indicated times. Rice protoplasts were treated with 100 µM C6-ceramide (C6-cer) or C6-dihydroceramide (C6-DHC) under light. The viability was determined by FDA staining. Standard errors in (A) and (B) are shown (n = 3). Letters indicate that values of viability differed in Fisher's PLSD test, a post hoc multiple t test (P<0.05). Control treatment was with 0.15% ethanol (the solvent for C2 or C6). (C) In situ detection of DNA fragmentation by TUNEL after C6-ceramide treatment. Rice protoplasts were treated with 0.15% ethanol and 100 µM C6-ceramide for 12 h and stained using the TUNEL method as described in Materials and Methods. The green signal (arrow) indicates a TUNEL-positive nucleus. Protoplasts were stained for nuclei using DAPI. Scale bars represent 10 µM. (D) The DNA ladder induced by 100 µM C6-ceramide after 12 h treatment. (TIF) [file pone.0018079.s002.tif]
