# Supplementary material for: A Conserved Cysteine Motif Is Critical for Rice Ceramide Kinase Activity and Function
Source: PLoS One. 2011 Mar 31;6(3):e18079. doi: 10.1371/journal.pone.0018079 (PMC3069040; doi:10.1371/journal.pone.0018079)
Supplement: Methods S1 — Includes appendixes. (DOC) [file pone.0018079.s003.doc]

**Supplementary information**

**Appendix S1 Sequence alignment**

Based on the GenBank BLASTp results of OsCERK, we removed incomplete and hypothetical sequences, selected representative sequences according to the taxonomic groups, and aligned the remaining sequences using multiple alignment of ClustalW [1].

**Appendix S2 Rice protoplast and DNA isolation**

Rice protoplasts were isolated from 10-day-old seedling tissues as described previously with modifications [2]. We stripped the coleoptiles, cut etiolated young seedlings into approximately 0.5 mm strips and placed these in baffled flasks containing 0.6 M mannitol for 10 min. The chopped tissues were then transferred to an enzyme mixture [1.5% (w/v) cellulase RS and 0.75% (w/v) macerozyme R10 (Kinki Yakult, Tokyo, Japan), 10 mM MES (pH 5.7), 0.1% (w/v) bovine serum albumen, 1 mM CaC1_2_, 5 mM-mercaptoethanol and 0.6 M mannitol] and shaken at low speed at room temperature for 3-4 h. Protoplasts were collected with a 40 μm nylon mesh and washed in the W5 solution (154 mM NaCl, 125 mM CaCl_2_, 5 mM KCl, 2 mM MES, 5 mM glucose adjusted to pH 5.7 with KOH). The viability of protoplasts after treatments was determined using FDA staining using a hemocytometer and a fluorescence microscope with Zeiss filter set 38 (Axio Imager A1, Carl Zeiss).

DNA isolation was performed as described previously [3] with several changes. The protoplasts were lysed in lysis buffer [750 μl 0.1 M Tris-HCl (pH 7.5), 50 mM EDTA, 0.5 M NaCl and 100 μl 10% SDS] at room temperature for 15 min, and then we added 250 μl CTAB buffer (0.2 M Tris-HCl pH 7.5, 50 mM EDTA, 2 M NaCl and 2% CTAB) incubated for 15 min at 65 ^o^C. The final ethanol precipitation of DNA was followed by digestion using RNase at 100 μg/ml for 1 h at 37 ^o^C. Identical amounts of DNA were separated on a 2% agarose gel.

For LM-TUNEL assay, treated protoplasts were ﬁxed in 4% paraformaldehyde for 1 h at room temperature. The free 3′-OH groups in the DNA were labeled by the TUNEL method using the ApopTag^R^ Plus Fluorescein *in situ* Apoptosis Detection Kit (CHEMICON, Catalog number S7111, USA) as described previously [4].

**Appendix Accession numbers**

Sequence data of the analyzed CERK proteins in this article can be found in the GenBank data library under the following accession numbers. *Homo Sapiens* (GenBank accession number NP_073603.2), *Mus musculus* (GenBank accession number NP_663450.3), *Rattus norvegicus* (GenBank accession number NP_001128333.1), *Canis familiaris* (GenBank accession number XP_531694.2), *Equus caballus* (GenBank accession number XP_001488792.1), *Ornithorhynchus anatinus* (GenBank accession number XP_001506327.1), *Monodelphis domestica* (GenBank accession number XP_001375028.1), *Gallus gallus* (GenBank accession number NP_001026511.1), *Danio rerio* (GenBank accession number NP_001099056.1), *Nasonia vitripennis* (GenBank accession number XP_001600118.1), *Tribolium castaneum* (GenBank accession number XP_969216.1), *Drosophila melanogaster* (GenBank accession number NP_730923.1), *Culex quinquefasciatus* (GenBank accession number XP_001845068.1), *Aedes aegypti* (GenBank accession number XP_001649407.1), *Caenorhabditis elegans* (GenBank accession number AAC67466.1), *Brugia malayi* (GenBank accession number XP_001901468.1), *Vitis vinifera* (GenBank accession number CAO60836.1), *Populus trichocarpa* (GenBank accession number EEE77479.1), *Arabidopsis thaliana* (GenBank accession number AY362552), *Physcomitrella patens* subsp. *patens* (GenBank accession number XP_001761341.1). The analyzed DAGK proteins are *Chlamydomonas reinhardtii* (GenBank accession number XP_001694757.1) and *Exiguobacterium sibiricum* 255-15 (GenBank accession number YP_001813428.1). The analyzed SPK proteins are *Schizosaccharomyces pombe* (GenBank accession number NP_593818.1), *Saccharomyces cerevisiae* (GenBank accession number NP_013361.1) and *Dictyostelium discoideum* AX4 (GenBank accession number XP_645126.1). The GenBank accession number for the nucleotide sequence of OsCERK is FJ765452.

**References**

1. Thompson JD, Higgins DG, Gibson TJ (1994) CLUSTAL W: improving the sensitivity of progressive multiple sequence alignment through sequence weighting, position-specific gap penalties and weight matrix choice. Nucleic Acids Res 22: 4673-4680.

2. Bart R, Chern M, Park CJ, Bartley L, Ronald PC (2006) A novel system for gene silencing using siRNAs in rice leaf and stem-derived protoplasts. Plant Methods 2: 13.

3. Wang M, Oppedijk BJ, Lu X, Van Duijn B, Schilperoort RA (1996) Apoptosis in barley aleurone during germination and its inhibition by abscisic acid. Plant Mol Biol 32: 1125-1134.

4. Yao N, Tada Y, Park P, Nakayashiki H, Tosa Y, et al. (2001) Novel evidence for apoptotic cell response and differential signals in chromatin condensation and DNA cleavage in victorin-treated oats. Plant J 28: 13-26.
